# Adapting a model of response to child abuse to the conditions in the circumpolar north

**DOI:** 10.3402/ijch.v75.32713

**Published:** 2016-12-09

**Authors:** Lene Mosegaard Søbjerg, Alice Fredsgaard Thams

**Affiliations:** 1 VIA Society and Social Work, VIA University College, Aarhus, Denmark; 2 Education of Social Work, VIA University College, Holstebro, Denmark

Sexual and reproductive health is dependent on sexual well-being. Research shows that sexual abuse during childhood has long-term consequences on both mental and physical health of the victims during the rest of their lives (1). Furthermore, research shows that the children of mothers who themselves were victims of childhood abuse suffer from greater psychosocial maladjustment than other children (2). Numerous reports argue that social problems including sexual abuse of children are widespread in the arctic north (3). Preventing sexual abuse as well as dealing adequately with cases of abuse is of utmost importance to ensure sexual and reproductive health. The methods and models of dealing with cases of child abuse consequently play an important role in the promotion of sexual and reproductive health (1).

In 2010, Naalakkersuisut, the Government of Greenland, decided to establish a centre to deal with cases of child abuse. Saaffik (Saaffik.gl) was inspired by the Children's Advocacy Center (CAC) developed in the United States in the 1980s. The CACs represent a child-friendly, multi-disciplinary response to child abuse with the dual intention of facilitating the legal process and ensuring that the child victims receive the necessary support (www.nationalcac.org).

Saaffik was opened in 2011 in the capital of Greenland, Nuuk. Based on the principles of CAC and the Scandinavian-adopted models called “barnahus” (4), Saaffik had a “one-door” approach which provided a coordinated response to the child victims and ensured that relevant institutions and authorities co-operated. The multidisciplinary approach ensured that children could meet all relevant professionals within Saaffik, including medical staff, police and social services. An example of the inclusion of multiple professions was the furnishing of a room to accommodate video recording of police interviews.

The model and idea of the “one-door” principle is compelling and has had positive impact on the treatment of child abuse in the United States and countries in Scandinavia (5). However, in Greenland the implementation of a “one-door” model for the entire country is challenged. The geography and the enormous distances in Greenland make it impossible to secure the transportation of children from all of Greenland to Nuuk. Lack of educated and trained staff makes it difficult to meet the needs of children across the country. In fact, during the 5 years, Saaffik has existed, and it has almost exclusively dealt with children from Nuuk.

Currently, the structure of Saaffik is undergoing changes. Saaffik is no longer an independent centre but part of the Central Advisory Unit under the Department of Children and Families, established to assist municipalities and social workers in their work with vulnerable children. Saaffik remains a child-friendly place with knowledge and expertise on child abuse, but the “one-door” model is abandoned. The police no longer conducts video-interviews at Saaffik, and medical examinations take place at hospitals and medical centres. Instead of the “one-door” principle, Saaffik now concentrates on becoming a knowledge centre, with expertise to help social workers throughout Greenland in their dealing with child abuse. Furthermore, a travelling team of experts has been established. The travelling team includes therapists, psychologists and social workers. The responsibility of the travelling team is to assist and help authorities and victims of abuse. The working method is to reach the victims where they live and initiate therapy and social- and health-related efforts to help them rehabilitate. Another advantage of the travelling team is that in the small villages in Greenland everyone knows each other, which makes it difficult for the abused child to trust a local professional, who knows the abuser personally. It is easier for the abused children to trust the professionals in the travelling team because they have no personal relations with the abuser.

The development away from the “one-door” model and towards an out-reaching travelling team seems to be a productive way of providing adequate assistance to child victims of sexual abuse in Greenland. The strengthening and centralisation of expertise combined with the ambition to meet the children where they live is an example of using inspiration from an existing model and transforming it into a model more suitable for the circumstances of life in the circumpolar north. The new developments at Saaffik provide positive aspirations of establishing an institution, which can effectively deal with the consequences of sexual abuse. With adequate resources and political support, the developments of Saaffik have the potential for improving the sexual and reproductive health of children in Greenland. Furthermore, an increased focus on the problems of sexual abuse may improve the public health and well-being of the entire population of Greenland as the long-term consequences of abuse are reduced.

An important lesson of the case of implementing an American/Scandinavian model of handling child sexual abuse in Greenland is that simply adopting a foreign model does not necessarily meet the needs of the circumpolar territories. Finding inspiration in models from other countries certainly makes sense, but it is vital to adapt and adjust a model to the needs and circumstances of the populations in question.
